# Attention‐gated U‐Net networks for simultaneous axial/sagittal planes segmentation of injured spinal cords

**DOI:** 10.1002/acm2.14123

**Published:** 2023-09-21

**Authors:** Nicolas Masse‐Gignac, Salomón Flórez‐Jiménez, Jean‐Marc Mac‐Thiong, Luc Duong

**Affiliations:** ^1^ Department of software and IT engineering École de technologie supérieure Montréal Canada; ^2^ Department of orthopedic surgery Hopital Sacré‐Coeur Montréal Canada

**Keywords:** convolutional neural networks (CNN), deep learning, medical image segmentation, spinal cord

## Abstract

Magnetic resonance imaging is currently the gold standard for the evaluation of spinal cord injuries. Automatic analysis of these injuries is however challenging, as MRI resolutions vary for different planes of analysis and physiological features are often distorted around these injuries. This study proposes a new CNN‐based segmentation method in which information is exchanged between two networks analyzing the scans from different planes. Our aim was to develop a robust method for automatic segmentation of the spinal cord in patients having suffered traumatic injuries. The database consisted of 106 sagittal MRI scans from 94 patients with traumatic spinal cord injuries. Our method used an innovative approach where the scans were analyzed in series under the axial and sagittal plane by two different convolutional networks. The results were compared with those of Deepseg 2D from the Spinal Cord Toolbox (SCT), which was taken as state‐of‐the‐art. Comparisons were evaluated using K‐Fold cross‐validation combined with statistical *t*‐test results on separate test data. Our method achieved significantly better results than Deepseg 2D, with an average Dice coefficient of 0.95 against 0.88 for Deepseg 2D (*p* <0.001). Other metrics were also used to compare the segmentations, all of which showed significantly better results for our approach. In this study, we introduce a robust method for spinal cord segmentation which is capable of adequately segmenting spinal cords affected by traumatic injuries, improving upon the methods contained in SCT.

## 1 INTRODUCTION

The spinal cord is a highly complex anatomical structure that is, part of the central nervous system. Its main function is to ensure the conduction of motor signals from the brain to the peripheral nervous system and the conduction of sensory signals from the peripheral nervous system to the brain. The degeneration of the spinal cord due to various diseases as well as the damage caused by traumatic accidents can therefore drastically alter the functioning of the human body.^[^ ^1^ ^]^ Magnetic resonance imaging is the modern tool of choice for evaluating spinal cord injuries.^[^ ^2^, ^3^, ^4^, ^5^, ^6^ ^]^ Segmentation of the spinal cord by MRI is most often used to measure its cross‐sectional area at various levels, which allows quantitative analyzes to be carried out in order to characterize lesions.^[^ ^7^, ^8^ ^]^ Automatic segmentation of the spinal cord is also used to build standardized atlases such as in the Spinal Cord Toolbox^[^ ^9^ ^]^ (SCT) and can facilitate longitudinal studies where manual segmentation is too time‐consuming to accomplish.

Several approaches exist for the automatic segmentation of the spinal cord from MRI. Some methods, such as Propseg,^[^ ^10^ ^]^ use algorithmic approaches without neural networks, while more recent approaches such as Deepseg 2D (SCT), Deepseg 3D^[^ ^11^ ^]^ or BASICseg^[^ ^12^ ^]^ use deep learning methods based on U‐Net type architectures.^[^ ^13^, ^14^ ^]^ According to the authors presenting Deepseg 3D, their approach is able to segment healthy spinal cords or those with non‐traumatic lesions with high precision on T2‐weighted scans (Dice coefficient > 0.92).^[^ ^11^ ^]^ However, the quality of segmentations in patients with traumatic spinal cord injury (SCI) is considerably lower, with Dice coefficients of 0.70 for spinal cord areas affected by traumatic lesions and 0.90 for whole spinal cord segmentations.^[^ ^12^ ^]^ This is because the normal features of the structures surrounding the spinal cord in the injured areas are lost or distorted. Deepseg approaches also have difficulty segmenting the spinal cord of some older subjects, since the demarcations surrounding it may be less clear.

The objective of this study is to develop an approach for injured spinal cord segmentation that is, versatile enough to be used in a clinical setting. This method uses T2‐weighted sagittal scans of injured spinal cords to train two convolutional networks placed in cascade. The scans are first sent to one network, which analyzes them in the axial plane and produces a primary segmentation. This segmentation along with the original scan is then fed to the second network, which analyzes them in the sagittal plane and produces the final segmentation. Thus, we have developed a robust method capable of segmenting not only healthy spinal cords, but also those with traumatic injuries or degraded by age. To this end, an automatic method incorporating the standardization of MRI data as well as the training of convolutional networks has been developed. The networks are evaluated using several metrics, including overlap, statistic and distance metrics. The results are compared to those of the Deepseg 2D network from SCT (version 5.6), which represents the state of the art for our data, as testing our data with other available approaches from SCT such as Deepseg 3D rendered significantly inferior results.

## 2 MATERIALS AND METHODS

### 2.1 Data

The study population consisted of patients admitted to the Sacré‐Coeur Hospital of Montreal after having suffered traumatic spinal cord injuries. A total of 106 sagittal T2 weighted scans were used, sourced from 94 patients. Most scans were cervical (94), with some patients also having dorsal (8) or lumbar (4) scans. Most scans were taken on Siemens machines, with 59 scans from the MAGNETOM Avanto model (1.5 T), 27 scans from the MAGNETOM Symphony model (1.5 T) and 19 scans from the MAGNETOM Skyra model (3 T). One scan from was from the Philips Achieva model (3 T). Table 1 shows the detailed scan parameters for the entirety of our dataset.

**TABLE 1:** Overview of scan parameters.

Parameters	MAGNETOM Avanto	MAGNETOM Symphony	MAGNETOM Skyra
Sagittal resolution (mm)	0.488 [0.528, 0.684]	0.656 [0.586, 0.703]	0.651 [0.488, 0.684]
Slice gap (mm)	3.4 [3.3, 4.8]	3.4 [3.3, 4.8]	3.5 [3.3, 5.0]
Slice thickness (mm)	3.03 [3.0, 4.0]	3.04 [3.0, 4.0]	3.05 [3.0, 4.0]
Repetition time (s)	3.78 [2.97, 4.99]	3.52 [2.80, 4.71]	3.38 [2.74, 4.00]
Echo time (s)	0.111 [0.104, 0.158]	0.125 [0.118, 0.132]	0.100 [0.081, 0.103]
SAR (W/kg)	0.993 [0.644, 2.359]	2.026 [1.372, 3.284]	0.704 [0.393, 1.441]
Flip angle (°)	155.7 [150.0, 180.0]	170.4 [153.0, 180.0]	159.5 [150.0, 160.0]
FOV (%)	100.0 [100.0, 100.0]	100.0 [100.0, 100.0]	100.0 [100.0, 100.0]
Echo train length	18.0 [15.0, 29.0]	21.2 [15.0, 23.0]	18.0 [11.0, 19.0]
Phase encoding steps	601.5 [451.0, 648.0]	486.8 [437.0, 615.0]	497.05 [361.0, 717.0]
Acquisition matrix PE	300.0 [246.0, 326.0]	256.8 [238.0, 307.0]	281.4 [250.0, 358.0]
Number of scans (field)	59 (1.5T)	27 (1.5T)	19 (3T)

For manual segmentation of the whole spinal cord, the segmentations from Deepseg 2D were used to create preliminary templates. These templates where then annotated manually by a trained rater. A fellow in neurosurgery (S.F.J.)subsequently reviewed the manual segmentations and also produced segmentations of the spinal cord injuries themselves. In addition to the T2 scans being segmented, most patients possessed axial scans as well as T1 and STIR scans which were sometimes used to clarify ambiguous areas.

The data was randomly separated into training and test data. The data from 19 patients, or 20 scans, were reserved for testing, while data from the remaining 75 patients, or 86 scans, were dedicated to training. We thus obtained a training/test separation of approximately 80/20. Since many patients had more than one scan, it was important to ensure that all scans for the same patient were in only one of the two categories in order to avoid biasing the results in a favorable way.

For manual segmentation of the whole spinal cord, the segmentations from Deepseg 2D were used to create preliminary templates. These templates where then annotated manually by a trained rater. A fellow in neurosurgery [name deleted] subsequently reviewed the manual segmentations and also produced segmentations of the spinal cord injuries themselves.

The data was randomly separated into training and test data. The data from 19 patients, or 20 scans, were reserved for testing, while data from the remaining 75 patients, or 86 scans, were dedicated to training. We thus obtained a training/test separation of approximately 80/20. Since many patients had more than one scan, it was important to ensure that all scans for the same patient were in only one of the two categories in order to avoid biasing the results in a favorable way.

### 2.2 Segmentation framework

**FIGURE 1:**
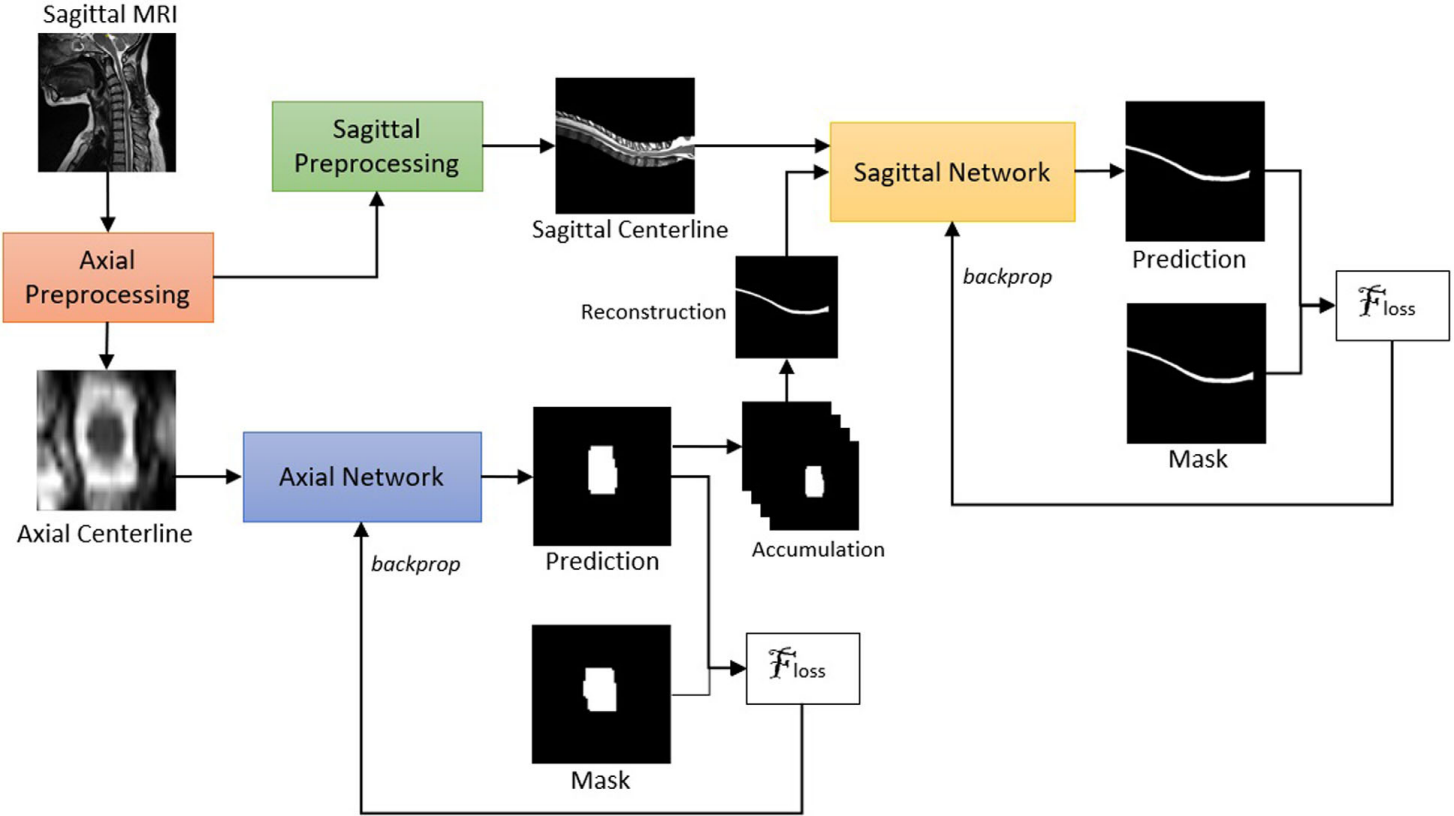
Overview of our approach during training. The first step consisted in training the axial network. Following this, the segmentations produced were reconstructed sagittally and used in pair to train the sagittal network. When using this method for inference on a given scan, all axial slices of the scan are first segmented and then rearranged to form a sagittal segmentation, which is fed to the sagittal network along with the sagittal centerline to produce the final segmentation.

In our approach, one convolutional network first segmented all of the axial slices of a scan. Following this, the axial segmentations produced were rearranged to form sagittal slices and forwarded, along with the sagittal MRI slices, to another convolutional network to be analyzed sagittally. Our approach, which targeted the segmentation of the entire spinal cord, was therefore divided into two stages, as can be seen on Figure 1. The first stage consisted in training a convolutional network using axial slices in order to produce a preliminary segmentation of the spinal cord. This step mirrored the approach of BASICseg^[^ ^12^ ^]^ in the sense that it only used axial slices. However, the BASICseg approach used only axial type scans and analysed these in the axial plane only, while our approach used sagittal scans and analyzed these in the axial as well as the sagittal planes.

The second step of this approach used as input data the sagittal slices of the scans as well as the rearranged axial segmentations obtained during the first step. Its objective was to train a second convolutional network using this data, this one to segment the sagittal slices. This combination of axial and sagittal information afforded the second network a more complete contextual representation of the information contained in the scans.

**FIGURE 2:**
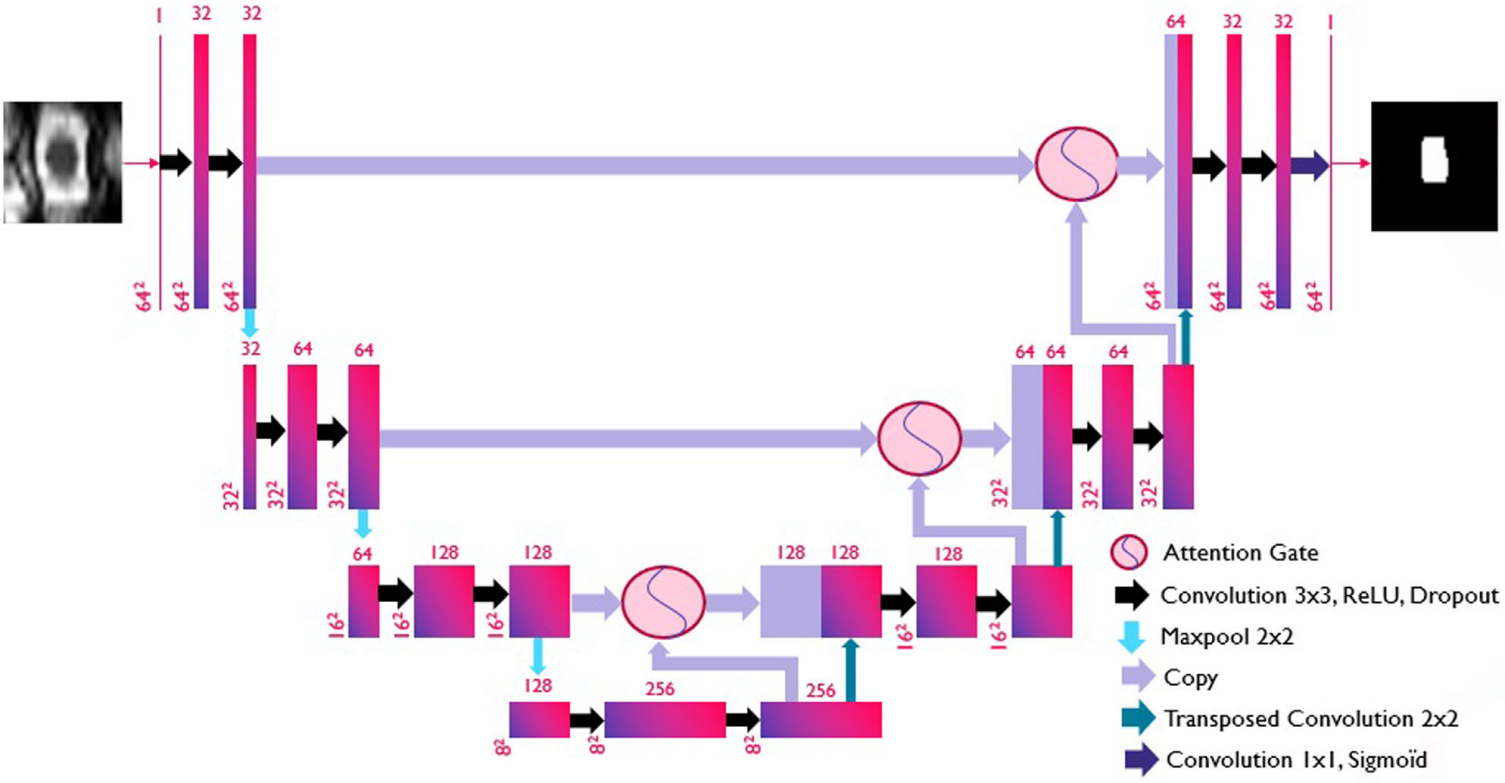
Architecture of our axial network. Note that the sagittal network used the same basic configuration, but with two input channels instead of one and larger feature map sizes.

The same architecture was used for both the axial and sagittal networks. This architecture, which is illustrated on Figure 2, is based on the original U‐Net^[^ ^15^ ^]^ and preserves the contracting and expanding paths principle as well as the skip connections between the two paths. However, several adaptations to the original architecture have been made.

The number of feature maps produced in the initial convolution was reduced from 64 to 32. This speeds up the network considerably, reducing by half the number of feature maps in the entire network while maintaining performance for our task. We also reduced the contracting/expanding layers from four to three, again improving efficacy. In our case, the extra layer not only slowed down the network, but had a detrimental effect on performance. This was probably due to excessive deterioration of feature map resolution in the lower layers,^[^ ^16^ ^]^ especially for our axial network, since initial slice resolution was low. We also added a zero‐padding of 1 × 1 to all convolutions, thus keeping feature map sizes constant in each layer and eliminating the need for cropping prior to concatenation of the skip connections.

Another change made to the original U‐Net architecture was to add dropout layers^[^ ^17^ ^]^ with a coefficient of 0.4 following each ReLU layer. This improved performance considerably by reducing overfitting and thus allowed training to be prolonged. A sigmoïd layer was also added as the final layer in our network in order to bring the output values between 0 and 1.

The final change from U‐Net was to add attention gates^[^ ^18^, ^19^ ^]^ preceding the concatenation of skip connections. These attention gates help to transmit the more global but lower resolution information from the deeper layers to the higher layers. Our preliminary experiments showed an improvement in performance when these gates were used, and since they did not add considerable weight to the network, we choose to include them in our final network.

Upsampling in the expansive path was achieved using unpadded 2 × 2 transposed convolutions with a stride of two. This restored all feature maps to the exact size of the next layer in the expansive path, preventing the need for any cropping or superfluous operations.

The axial and sagittal networks differ in only two respects. First, the sizes of the feature maps differ at each layer. For the axial network, they are successively 64^2^, 32^2^, 16^2^, and 8^2^ in the contracting path, while for the sagittal network, they are 384^2^, 192^2^, 96^2^, and 48^2^. Additionally, in the case of our sagittal network, there are two input channels rather than one, as this network takes as simultaneous input sagittal centerlines and segmentations from the axial network.

#### 2.2.1 Preprocessing

**FIGURE 3:**
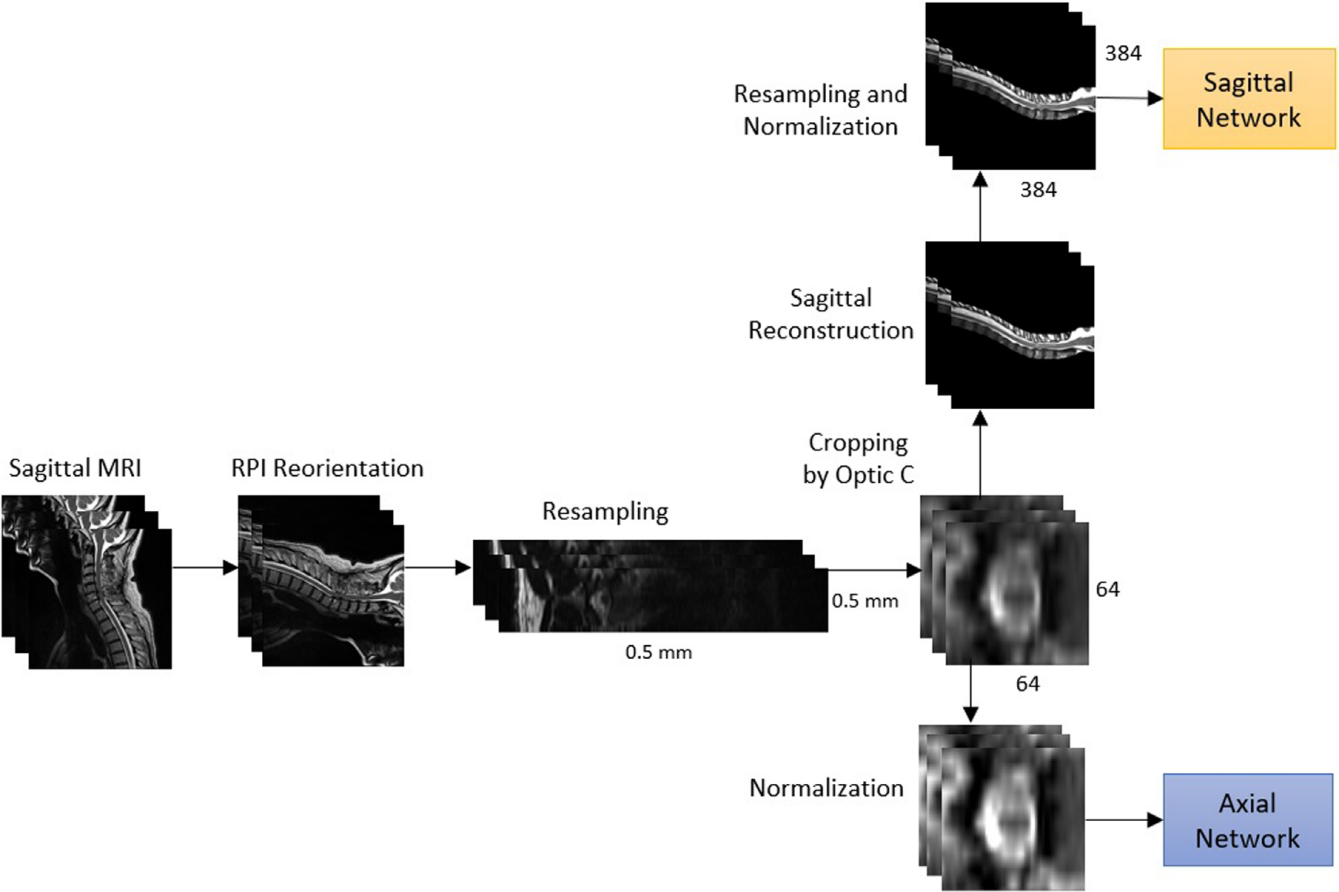
Preprocessing overview of our approach. Steps common to the preprocessing of both networks are represented horizontally while steps specific to each network are represented vertically.

All scans were firstly re‐orientated and re‐sampled to 0.5 mm in the axial plane. Following this, we used the Optic C algorithm^[^ ^20^ ^]^ from SCT in order to isolate a 32 mm × 32 mm area around the spinal cord centerline, which was equivalent to 64 × 64 voxels. Subsequently, an intensity normalization was applied to the axial slices by limiting the maximum and minimum intensities to correspond with the 98th and 2nd intensity percentiles of the original image. The range of values for the voxels was also standardized through linear interpolation to values between 0 and 1. The axial slices obtained were then forwarded to the axial network for training or inference. For the sagittal network, the axial centerlines were used along with spatial information stored during their cropping to construct sagittal centerlines over a black background. Finally, the sagittal centerlines were re‐sampled to dimensions 384 × 384 voxels and normalized using the same process as the axial centerlines before being forwarded to the sagittal network. An overview of the preprocessing steps is illustrated on Figure 3.

#### 2.2.2 Augmentation

Elastic transformations and rotations were used in order to prevent overfitting and increase the capacity of the networks to analyze new data. Because subjects are not always scanned in exactly the same position, there are slight variations as to the angle of their spinal cord in relation to the image borders. The rotations therefore simulate situations where the subjects' positions would have been slightly different from their actual position, thereby increasing the variety of the data set. As for the elastic transformations, these simulate scenarios where the patients would have different spinal curvatures. The use of these augmentations was established by comparing preliminary results using different augmentation types and coefficients, and then finally selecting the most promising values.

These augmentations were realized by applying identical operations to the MRI slices and their corresponding masks. The augmentation was reapplied at certain epochs of training, using random coefficients over a specified range to generate new transformations. In this way, novel information was fed to the networks periodically. The unmodified slices were also included in the training data, so that at any given moment during training, half of the training data consisted of the original slices, while the other half consisted of augmented data. In this way, the size of the original training set was doubled.

#### 2.2.3 Postprocessing

No postprocessing was applied to the segmentations during training. At inference‐time, a simple postprocessing was applied to the segmentation mask to ensure that it contained a minimum of 20 voxels.

## 3 EVALUATION AND RESULTS

### 3.1 Evaluation

This approach was realized using the Python programming language (version 3.6.9) in conjunction with the deep learning library PyTorch (version 1.9.0). The networks were trained on the Colaboratory platform using a NVDIA Tesla P100 GPU.

#### 3.1.1 Cross‐validation

Training was performed following the K‐Fold cross‐validation process, with K = 10. During this process, the training database was randomly divided into 10 distinct validation subsets. Each of these validation subsets had its own scans, which were not shared with any other subset. Out of a total of 86 scans reserved for training, eight or nine of them were therefore allocated to each validation subset, while the remaining scans were used to train the networks themselves. Out of 10 subsets, there were therefore six subsets which had nine scans reserved for validation and four subsets which had eight.

Statistical analysis was performed for each of the 10 networks in order to produce *p*‐values comparing them to Deepseg 2D. In this process, each network was first used to segment the scans in our test database (*n*=20). Deepseg 2D was also used to segment these scans. At this stage, an Agostino‐Pearson test was used to establish the normality of each result population. Next, an error matrix was produced for each of our 10 networks by subtracting their result matrices with that of Deepseg 2D. From these error matrices, we used a paired *t*‐test with 19° of freedom in order to obtain a t‐value for each of our 10 cross‐validation networks.

*p*‐values were then calculated from these t‐values using a two‐tailed hypothesis. The null hypothesis implied by this process was that the mean error values for each network and Deepseg 2D were equal. The *p*‐values produced therefore represented the probability of our networks and Deepseg 2D having the same mean error on the test data, so that smaller *p*‐values indicated a more significant difference in network performance.

This way of proceeding was statistically valid,^[^ ^21^, ^22^ ^]^ since our networks and Deepseg 2D were trained with independent data. It is however important to keep in mind that data from our clinical site was used for training our networks as well as for these comparisons.

#### 3.1.2 Axial training

There were a total of 39,728 axial slices contained in the 86 training scans. We therefore obtained an average training/validation separation of 35,755/3973 slices across the 10 cross‐validation subsets. As all scans did not have the same dimensions, and as the validation subsets did not contain the same number of scans, the proportion of axial slices dedicated to validation and training for each subset varied slightly.

During training, each axial slice was augmented with an elastic coefficient chosen randomly between $-10^{\circ }$ and 10° as well as a rotation coefficient between $-5^{\circ }$ and 5°. Unmodified slices were also kept in the training set, so as to double the quantity of axial slices dedicated to training. In addition, augmented slices were renewed at each epoch so as to maintain a constant supply of new data to the network, as discussed previously.

**FIGURE 4:**
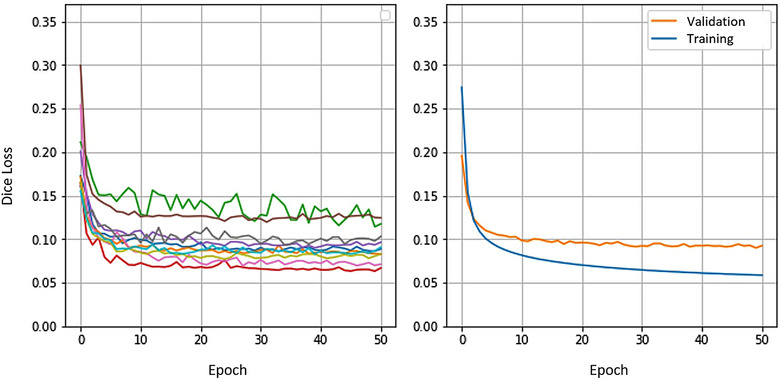
Validation loss curves for each of the 10 cross‐validation subsets (left) and average training and validation losses for all subsets (right). Average validation losses reach minimum value at epoch 49.

Following the augmentation, the training subsets ended up with an average of 71,510 axial slices. The networks were then trained for 50 epochs or 893,900 iterations, which was sufficient for the validation loss curves of the subsets to flatten out. This is usually when networks begin to overfit, especially when training losses continue to decline. This flattening phenomenon is clearly visible in Figure 4, which shows the average of the validation losses for all the subsets.

Training parameters were selected by using a process based on genetic selection, and included a batch size of 4, a dropout rate of 0.4 and a learning rate of 5e‐5. We used Dice Loss^[^ ^23^ ^]^ and the Adam optimizer^[^ ^24^ ^]^ with betas equal to (0.5, 0.999) for backpropagation.

#### 3.1.3 Sagittal training

Once the axial networks were fully trained, they were used in turn to segment all of the training and validation scans. The segmentations produced were then reconstructed sagittally and matched with their corresponding sagittal centerlines in preparation for training the sagittal network. Note that the same training/validation separations that were used for each cross‐validation iteration of the axial training were maintained. The various parameters used during the training process were again selected following an optimization process based on genetic selection. The values retained included a batch size of 2, a dropout rate of 0.4 and a learning rate of 5e‐5. The same loss function and optimizer were used as in the axial training.

There were a total of 1177 sagittal slices contained in the 86 training/validation scans. However, only slices whose segmentations by the axial network contained segmented voxels were kept for training. This way of proceeding was found experimentally and greatly reduced the number of sagittal slices used. In effect, by eliminating slices that did not contain spinal cord voxels, each scan was reduced to 4 or 5 slices rather than 13 or 15. This allowed the network to focus only on relevant slices, thus avoiding wasting resources at attempting to identify the characteristics of slices that did not contain spinal cord voxels. It should however be noted that following the training, during the inference on the test data, all of the slices were sent to the network. As we will see later, the sagittal networks therefore had the ability to recognize empty slices even if these were not used during training.

Following this slice reduction, we obtained an average training/validation separation of 348/39 sagittal slices for the 10 cross‐validation subsets. As was the case during axial training, since the scans did not all have the same dimensions and the validation subsets did not all have the same number of scans, the proportion of sagittal slices dedicated to validation and training for each subset varied slightly.

During training, each sagittal slice was augmented with a randomly chosen elastic coefficient between $-50^{\circ }$ and 50°, and a random rotation coefficient between $-15{}^{\circ }$ and 15°. The unmodified slices were also kept in the training set, doubling the number of sagittal slices dedicated to training. The augmented slices were renewed at an interval of 10 epochs, in contrast with the axial training, which renewed the augmented slices at each epoch. This way of proceeding was found experimentally. Following this augmentation, the training subsets ended up with an average of 696 sagittal slices. The networks were then trained for 125 epochs or 43,500 iterations, which once again corresponded to the moment when the validation losses flattened out while the training losses continued to decline.

### 3.2 Results

The Dice coefficient is well suited for evaluating segmentations where the classes of an image are imbalanced,^[^ ^23^, ^25^, ^26^, ^27^ ^]^ which is frequently the case for medical images. Since the volume of the spinal cord in our scans was very small relative to the total volume of the scans, our images were no exception. Table 2 shows that the mean Dice coefficient for our 10 cross‐validation networks is 95.3, while the Deepseg mean is 87.9 (*p* < 0.001). Additionally, all 10 networks taken individually had Dice coefficients higher than Deepseg with *p*‐values smaller than 0.001. It can therefore be concluded that our networks showed a significant improvement in regards to this metric.

**TABLE 2:** Final results for our approach. The scores for each metric represent the values obtained by comparing the segmentations of each network with the manual ground truth segmentations. The results represent the average of the 10 axial and combined networks over the test set. Bold values represent the best result for each metric.

	Axial	Combined	Deepseg
Dice coefficient	89.9	**95.3**	87.9
True positive rate	0.92	**0.94**	0.89
Mean surface distance	0.39	**0.13**	0.26
Hausdorff distance	1.53	**1.51**	2.01

The combined approach, in which the results from the axial networks were used to train the sagittal networks, also shows a marked improvement in comparison with results from the axial networks.

The true positive rate (TPR) is a statistical metric indicating the proportion of voxels segmented by the network that are also segmented on the mask. This metric is often used to measure the precision of segmentations.^[^ ^28^, ^29^ ^]^ All of our networks had a significantly higher TPR than Deepseg, with an average rate of 0.94, while Deepseg 2D had an average rate of 0.89 (*p* = 0.002). The axial networks' result for this metric are poor, indicating that the precision of these networks could be a point of weakness for them. This confirms the superiority of the combined approach, which improves upon the axial networks to render markedly superior TPR results.

The mean surface distance (MSD) measures the average Euclidean distance between pixels located on the contours of two sets. This metric is often used to evaluate the similarity between segmentations and their ground truth.^[^ ^16^, ^29^ ^]^ All of our networks had a significantly lower MSD than Deepseg 2D, with an average of 0.1319 against 0.2641 for Deepseg 2D (*p* < 0.001). The axial networks do not perform well in regards to this distance metric, obtaining an average MSD of 0.39, which demonstrates that the sagittal networks of the combined approach are instrumental in reducing this value.

The final metric used is the maximal Hausdorff distance (MHD), measures the maximum Euclidean distance from any point in a set to the closest point in another set. When calculating this metric, the two sets are evaluated in turn one against the other and only the higher of the two maximal distances found is retained. The MHD is frequently used to assess the quality of segmentations.^[^ ^16^, ^25^, ^29^ ^]^ All of our networks had a significantly lower MHD than Deepseg 2D, with an average of 1.51 against 2.01 for Deepseg 2D (*p* < 0.001). The axial networks perform well in regards to this metric, achieving an average MHD of 1.53. The sagittal step of the combined approach therefore does not seem to improve this metric, contratry to the three previous metrics.

**FIGURE 5:**
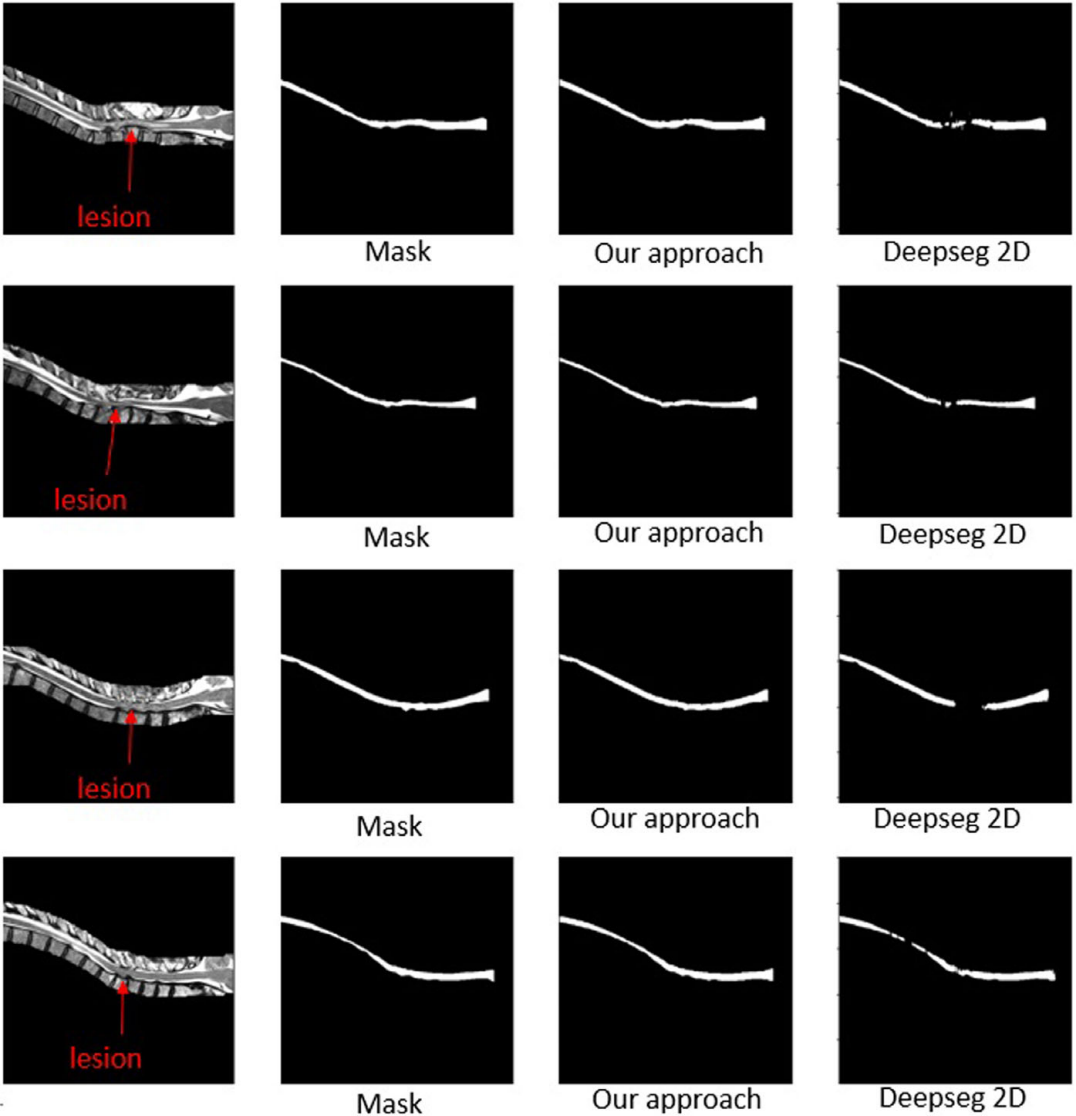
Visual comparison of our approach with Deepseg 2D. The images shown were chosen to represent typical differences between our segmentations and Deepseg's.

As can be seen in Figure 5, the majority of our segmentations appeared more uniform than those of Deepseg 2D, particularly in areas affected by spinal cord injuries. These areas are usually characterized by spinal cord discoloration, and areas of traumatic injury frequently lose their contrasting cerebrospinal fluid boundaries, making them even more difficult to segment. In these areas, the spinal cord often appears unrecognizable when viewing individual axial slices, because of the lack of information regarding the surrounding context. Likewise, it is also difficult ascertain spinal cord voxels in injured areas of sagittal scan due to lack of axial context.

By relaying the incomplete axial segmentations of the injured areas to the sagittal network, additional context is provided, helping the sagittal network identify spinal cord voxels that would previously have been impossible to categorize adequately due to lack of axial context. Combining the axial and sagittal is therefore the main strength of our approach, allowing for precise segmentation of spinal cords affected by traumatic injuries.

Another strength of our approach was its ability to segment sagittal slices that border the spinal cord. On these sections, the spinal cord most often appears only partially. The characteristic shape of the spinal cord is therefore lost, which makes sagittal segmentation more difficult. Axial slice, however, are not affected by this lack of context at the borders of the spinal cord. Our approach therefore here again takes advantage of contextual information sharing to improve segmentation of ambiguous areas.

Our approach thus appears to be significantly superior to Deepseg 2D for all metrics used as well as in visual comparisons. However, it is important to accompany these results with a few important points, which are discussed in the next section.

## 4 DISCUSSION

One consideration concerning the comparison of our approach with Deepseg 2D is the fact that the latter has not been exposed to data from our site while training. If we assume that data coming from one site is more homogeneous than data from multiple sites, this could put Deepseg 2D at a disadvantage during inference on data from our site. However, this disadvantage is difficult to quantify without data external to both approaches.

In terms of acquisition protocols, Deepseg 2D and our approach are equivalent, that is, both are designed to segment only T2‐weighted scans (the Deepseg 2D approach however contains other networks to segment T1 and T2* scans). Deepseg 2D and our networks are also both able to segment cervical and dorsal scans without discrimination and without loss of precision. The detection of the spinal cord centerline by the Optic C algorithm^[^ ^10^ ^]^ can be problematic for lumbar scans, however, since Deepseg 2D and our approach both use this algorithm, this fact does not prejudice the comparison.

Manual segmentations were adapted from Deepseg 2D segmentations. This may favor Deepseg 2D, as there are large sections of its segmentations that did not need to be touched up. In the study presenting the Deepseg 3D network,^[^ ^11^ ^]^ the authors employed the same methodology, using segmentations produced by Propseg as a basis to produce their manual segmentations. The authors of this article describe this as an advantage for Propseg when comparing it with their approach, which would reflect an advantage for Deepseg 2D during our approach.

As mentioned above, a simple postprocessing was applied to the segmentations at inference‐time, which consisted of erasing all of the segmented voxels on a given sagittal slice if these voxels numbered less than 20. Deepseg 2D uses more an extensive and complex postprocessing method, which includes filling of holes in the segmentations and preservation of only the largest object on each axial slice.

### 4.1 Future work

In the context of this study, our approach was limited to the analysis of sagittal scans. However, it would be interesting to develop an approach for axial scans using the same two‐plane technique. In this case, one possible approach would be to preserve the current structure, segmenting first axially and then sagittally. This way of proceeding would have the benefit of maintaining the sagittal network as the final output, allowing it to use its more global view of the spinal cord to provide longitudinal consistency.

Another possible approach for axial scans would be to first segment sagittal slices with lower resolution and then use the high resolution axial images for the final output. In this scenario, the final segmentations would benefit from the initial, lower‐quality sagittal segmentations while having sufficient high‐resolution information to produce precise final segmentations.

It is likely that a pair of combined axial/sagittal networks for each type of scan would perform better than a single pair combining axial and sagittal scans. Moreover, since it is possible to automatically detect the type of scan using information contained in their headers, it would be possible to create an automatic tool that would route the scans to the appropriate networks during inference. Such an approach would therefore have no foreseeable disadvantages compared to a mixed approach and would probably offer superior performance given the increased specificity of the networks.

## 5 CONCLUSION

The main objective of this study was to develop a tool capable of segmenting spinal cords affected by traumatic lesions. Our research led to a new approach using two attention‐gated U‐Net type networks in series to analyze MRI scans in the axial and sagittal planes conjointly. This approach was found to be significantly superior to the SCT's best approach for our data, Deepseg 2D, which was taken as the state‐of‐the‐art. The main contribution of this study is therefore the development of this strategy of combined networks, which is able to segment healthy spinal cords as well as instances with diseased or traumatic lesions.

Our method is therefore a promising lead not only for producing a spinal cord or spinal lesion segmentation tool which would be robust enough to be useful in a clinical setting, contrary to the tools currently available, but also for the analysis and segmentation of other anatomical structures captured by MRI.

This study built upon the work of the SCT, which is very effective for spinal cord segmentation in healthy patients. Traumatically injured spinal cords are however highly challenging and require a more robust segmentation method, with proper clinical annotation by experts. A method specifically tailed for the analysis of injured spinal cords, as proposed in this study, is therefore highly relevant for the clinical follow‐up.

## AUTHOR CONTRIBUTIONS

Nicolas Masse‐Gignac and Luc Duong designed and implemented the automatic segmentation technique. Jean‐Marc Mac‐Thiong provided feedback on clinical requirements including MRI caracteristics. Salomón Flórez‐Jiménez produced and reviewed the segmentation masks. All authors provided critical feedback, contributed to the research study design and contributed to the final manuscript.

## CONFLICT OF INTEREST STATEMENT

The authors have no relevant conflicts of interest to disclose.
